# COVID-19 Contact Tracing Apps: A Technologic Tower of Babel and the Gap for International Pandemic Control

**DOI:** 10.2196/23194

**Published:** 2020-11-27

**Authors:** Li Du, Vera Lúcia Raposo, Meng Wang

**Affiliations:** 1 Faculty of Law University of Macau Macau, SAR China; 2 Faculty of Law University of Coimbra Coimbra Portugal

**Keywords:** COVID-19, contact tracing apps, privacy, public health, global health

## Abstract

As the world struggles with the new COVID-19 pandemic, contact tracing apps of various types have been adopted in many jurisdictions for combating the spread of the SARS-CoV-2 virus. However, even if they are successful in containing the virus within national borders, these apps are becoming ineffective as international travel is gradually resumed. The problem rests in the plurality of apps and their inability to operate in a synchronized manner, as well as the absence of an international entity with the power to coordinate and analyze the information collected by the disparate apps. The risk of creating a useless Tower of Babel of COVID-19 contact tracing apps is very real, endangering global health. This paper analyzes legal barriers for realizing the interoperability of contact tracing apps and emphasizes the need for developing coordinated solutions to promote safe international travel and global pandemic control.

## Background

As the novel SARS-CoV-2 virus spreads worldwide—enabled by an unknown number of asymptomatic carriers—individuals continue to be at risk for potentially fatal infections. An effective method to contain the spread of the virus is tracing the movement of confirmed and suspected COVID-19 cases, as well as their close contacts [1]. To this end, many countries have used contact tracing mobile apps for controlling the COVID-19 epidemic. These apps are based on various techniques that can provide users with infection risk-level information specific to their communities and notify users who have been exposed to COVID-19, thus facilitating the public, social organizations, and the government to better prevent and control the epidemic [2].

With countries gradually reopening their borders for business and tourism, the effectiveness of using such apps for containing the spread of the pandemic has greatly weakened. The region-based development of contact tracing apps, along with the distinguished data protection laws used in different countries and regions, has resulted in the disconnection between contact tracing apps. These isolated contact tracing apps, which work as a technologic Tower of Babel, have lost their abilities to trace and monitor the spread of the pandemic in cross-border travel, triggering challenges for global pandemic control while international travel is resumed.

This viewpoint aims to raise global awareness on the urgent need for establishing a data sharing and transferring mechanism at the international level. It advocates that a consistent international effort should be devoted to developing an international data sharing platform for global pandemic control and removing the legal barriers that hinder the interoperability of contact tracing app technology. All these efforts are aimed to contribute a better response to potential future waves of COVID-19 and future global pandemic crises.

## Region-Based Contact Tracing App Development

COVID-19 contact tracing apps are designed and developed at the regional level, employing various technologies [3]. Asian countries, such as China, South Korea, and Singapore, are among the first group worldwide to develop and use tracking measures for fighting against the COVID-19 epidemic. In China for instance, the national government affairs service began developing the Epidemic Prevention and Health Information Code (EPHIC) after the pandemic surge in early February 2020 [4]. After a smart phone scans the EPHIC QR code, users submit their basic health status, residential addresses, and information about whether they have interacted with any confirmed or suspected individuals with COVID-19 within the past 14 days. With the spread of the COVID-19 pandemic, contact tracing apps have been widely used in many other countries.

Australia and New Zealand are also among the first countries that developed a national contact tracing app. For example, the COVIDSafe app launched by the Australian Department of Health helps state and territory health officials to identify any contact app users who have had close contact with patients with COVID-19 [5].

In Canada, contact tracing apps were first developed by provincial governments and used within their own territories. For example, the ABTraceTogether app in Alberta and the BC COVID-19 Support app in British Columbia both leverage epidemic tracking information provided by Canadian health departments to inform users about their epidemic exposure risks. The federal government also developed the COVID Alert app, which help users to better understand the risk levels of their own regions, as well as harness geographic location information to inform other users as to whether they have encountered anyone infected with SARS-CoV-2 [6].

Some states in the United States have developed contact tracing apps for local residents, such as the Care19 app in South Dakota and the Healthy Together app in Utah and Florida. Technology companies and research institutions have also taken up the challenge and have jointly developed software, such as the COVIDWatch app, NOVID app, and the Private Kit app. Through different technical means, such as self-reporting by users, Bluetooth, and GPS data, these apps can detect and record the contacts between app users, provide an indication of the risk of infection, and archive the data for the purpose of epidemic contact tracing [7]. In May 2020, Google and Apple jointly developed contact tracing technology and released application programming interfaces (APIs) that allow governments to work on developing their own contact tracing apps. Since then, more than one-fifth of the states in the United States have used the Google-Apple API code to develop their own contact tracing apps. To further promote the use of contact tracing technology, in September 2020, Google and Apple announced that they will incorporate their contact tracing tool into the latest operating system of their respective smartphones. This new system allows more public health authorities in the United States to embrace contact tracing functions, as no extra burdens are required to develop their own contact tracing apps [8].

In Europe, more than 20 countries have developed contact tracing apps for improving the management of COVID-19 [9]. Some countries, including France, Norway, and Hungary, have created their own model for contact tracing apps, while other countries, such as Germany, Netherland, and Switzerland, have used the Google-Apple API code to develop their contact tracing apps [10]. In June 2020, the UK government announced that it abandoned a centralized contact tracing app and adopted the new Google-Apple framework for future COVID-19 contact tracing software development to help achieve more effective pandemic control [11]. Initially, contact tracing apps were not welcomed by the European population or legal community, mostly due to privacy concerns and the requirements of the General Data Protection Regulation (GDPR). However, the second pandemic wave in Europe moderated the critics and highlighted the potential of contact tracing to control the progression of the virus.

## The Formation of the Technologic Tower of Babel

### Different Forms of Contact Tracing

Region-based contact tracing apps are developed by using diverse techniques (Table 1) and are used to collect different types of data. For instance, South Korea, New Zealand, Israel, and Taiwan have apps that transmit the users’ location. This technology model can allow the data subject to be identified by third parties, as demonstrated by an experience in South Korea, during which the disclosure of an infected individual’s location made it possible to identify the person in question [12]. Some governments force the installation of contact apps to monitor the flow of confirmed cases and travelers, while other jurisdictions, such as those in Singapore, Austria, and France, allow users to voluntarily choose to install the apps; users can also decide whether or not to have their data collected (ie, automatically collected via the app or by means of a voluntary act, such as reporting personal health information or by registering their location) [13]. Importantly, domestic and regional laws have different rules on data protection and privacy issues, which hamper transnational data sharing and exchange.

**Table 1:** Technologies used in COVID-19 contact tracing apps and potential legal challenges.

Technologies used for contact tracing apps	App examples, app name (country/region of origin)	Potential legal challenges
Location tracking by GPS	Self-quarantine safety protection (South Korea) Epidemic Prevention and Health Information Code (China) National Government Service Platform (China) HaMagen (Israel) NZ COVID Tracer (New Zealand) COVID Safe Paths (United States) Healthy Together (Utah, United States) Care19 (North Dakota, United States)	Personal privacy issues, including the disclosure of personal privacy location information, personal health information, etc [14]. It is not clear who will bear the responsibility if the users’ location is not accurate, the disease condition is reported incorrectly due to location information error, and there is a false report. If the state or the authorities adopt the technology on a large scale, but the technology fails due to satellite signals or technical failures, how can it be remedied? Some software shares data with the authorities, which could make it possible for the government to access personal location information. The issue is especially sensitive when it comes to the location of people of different races. Location can reveal a lot of personal information (eg, sexual orientation, religion, political affiliation) that is not directly related to pandemic control. With regard to apps that keep collected data in a central remote server, if the server is attacked, there will be a massive privacy breach.
Contact tracing by GPS	Epidemic Prevention and Health Information Code (China) National Government Service Platform (China) HaMagen (Israel) NZ COVID Tracer (New Zealand) COVID Safe Paths (United States) Healthy Together (Utah, United States) Care19 (North Dakota, United States)	All legal challenges for using GPS to track users’ location. Using GPS for contact tracing requires analyses of the location information of multiple users by means of big data. This involves methods for properly storing and using the personal information of users and other legal issues related to personal data privacy protection.
Contact tracing by Bluetooth	TraceTogether (Singapore) COVIDSafe (Australia) Stopp Corona (Austria) ABTraceTogether (Alberta, Canada) COVID Alert (Canada) ProteGO (Poland) Corona-Warn-App (Germany) SwissCovid (Switzerland) HaMagen (Israel) COVID Safe Paths (United States) Healthy Together (Utah, United States) Care19 (North Dakota, United States) CovidWatch (United States) NOVID (United States)	Although most of the software using Bluetooth technology has claimed that they will not obtain user information and will only warn people about the risk of disease through distance perception, data security is a big concern, as hackers may attack the Bluetooth firmware to obtain the user’s personal information and location data information [14]. Bluetooth technology faces many technical incompatibilities between devices, which will lead to incomplete information collection and, to some extent, the inability to effectively control the spread of the disease. Although privacy concerns remain, when Bluetooth relies on Bluetooth Low Energy technology, all information is stored in the user’s device (ie, a decentralized system), thus raising less privacy issues.
Self-reporting by users	Epidemic Prevention and Health Information Code (China) National Government Service Platform (China) Self-quarantine safety protection (South Korea) HealthLynked COVID-19 Tracker (United States) Relief Central COVID-19 (United States) PatientSphere for COVID-19 (United States) Obvio-19 (United States) How We Feel (United States) COVID Safe Paths (United States) CovidWatcher (New York City, United States) Care19 (South Dakota, United States) MyBellevue (Washington, United States) Healthy Together (Utah, United States) COVID Alert (Canada) BC COVID-19 Support (British Columbia, Canada) King’s College London Covid-19 Symptom Reporting (United Kingdom) NZ COVID Tracer (New Zealand)	Self-reporting software usually requires users to upload their own personal data. The software analyzes the location information and personal health data of multiple users by means of big data. This involves methods for properly storing and using the personal information of users and other legal issues related to personal data privacy protection. Under the absence of legal oversight, the self-reporting of health conditions by users can lead to the excessive collection of personal information by software. Centralized data storage may lead to the improper access of information and excessive dissemination of users’ personal information. Some self-reporting software shares data with the authorities, which can make it possible for the government to access personal information. There are no careful legal regulations to govern software and technology that collect data centrally for public health purposes in the context of pandemics. Who will be responsible for any false information? Will users that provide false information be held accountable?

### Different Jurisdictions Offer Different Ways to Protect Data

Existing domestic and regional data protection regulations are applicable for protecting users’ personal information, but their solutions are not always compatible (Table 2). For instance, within the European Union, apps are subject to the requirements of the GDPR [15], and eventually to those of Directive 2002/58/EC [16]. Data processing must comply with basilar general principles (Article 5/a-e of the GDPR) [17], as follows: (1) data must be collected in a fair way, and data processing must be open to public scrutiny, as per the principle of lawfulness, fairness, and transparency; (2) the collected data are to be used for a specific and clear purpose, and changes in purpose are not allowed, as per the principle of purpose limitation; (3) when possible, anonymized or pseudoanonymized data shall be used, and measures must be taken to prevent the reidentification of the data subject, as per the first dimension of the principle of data minimization; (4) unnecessary information is not to be collected, as per the second dimension of the principle of data minimization; (5) data must be accurate, and any mistake shall be easily amended, as per the principle of accuracy; and (6) data are to be stored for a limited period of time and destroyed after that, as per the principle of storage limitation.

**Table 2:** Examples of domestic and regional data laws applicable to data collection and data sharing of contact tracing apps.

Countries or regions	Applicable data laws
The United States	Health Insurance Portability and Accountability Act Health Insurance Portability and Accountability Act Privacy Rule California Consumer Protection Act
The European Union	General Data Protection Regulation Directive 2002/58/EC
Canada	Health Information Act Freedom of Information and Protection of Privacy Act
China	Cyber Security Law of the People’s Republic of China Personal Information Security Specifications
Australia	Privacy Amendment (Public Health Contact Information) Act

In the United States, although government-issued contact tracing apps exceed the scope of the Health Insurance Portability and Accountability Act (HIPAA) and HIPAA Privacy Rule, many contact tracing app developers claim that their apps will follow the requirements mandated by the HIPAA when collecting, storing, and using users’ data [18]. Some California-based companies further claim that their apps will also comply with the 2018 California Consumer Protection Act (CCPA) [19]. Even when only considering these 3 standards and disregarding all the remaining potentially applicable regulations, several different solutions can be found. Many of the rights guaranteed by the GDPR are unknown in US law, such as the right of data subjects to receive their personal data in a commonly used format and to transmit persona data to another data controller, the right to data portability, and the right to not to be subject to an adverse decision based solely on the application of artificial intelligence [20]. Moreover, the right to have one’s data erased upon request, which is a basic right under the GDPR, is absent from the HIPAA. However, for apps governed by the CCPA, this right will apply, since the right was established in Civil Code § 1798.105 [21].

In Canada, contact tracing apps developed by public health authorities are subject to the data protection of both the Health Information Act (HIA) and the Freedom of Information and Protection of Privacy Act (FIPPA). For example, the ABTraceTogether contact tracing app developed in Alberta must adhere to the privacy obligations of the HIA and FIPPA [22]. Thus, the app only collects the user’s nonidentifying information to trace the contacts of confirmed patients with COVID-19 [23]. In addition, based on the Public Health Act [24] and HIA [25], provincial health departments can track contacts during the COVID-19 pandemic and are able to use the information for health system management and planning, policy development, and public health emergency analysis. Unlike the GDPR, the FIPPA only applies to government institutions, and the protected personal information is narrower than the concept of personal data adopted by GDPR (eg, IP address and cookie data are not covered by the FIPPA) [26]. Moreover, provisions in the FIPPA [26] regarding data process and data subject rights are much less extensive than those speculated in the GDPR [27].

In China, the collection and processing of personal information garnered from COVID-19 tracking software is protected by laws and regulations that govern personal information protection and infectious disease prevention and control, such as the Cyber Security Law, the Personal Information Security Specifications, and the Regulations on Public Health Emergencies. Accordingly, when acquiring and using personal information, contact tracing apps shall obtain consent from the users in advance. The data processor shall also not intentionally disclose the personal information of confirmed or suspected patients with COVID-19 and their close contacts. Under the explicit authorization from users, disease prevention and control institutions and medical institutions can track high-risk populations based on legally obtained information. However, China’s data protection laws and regulations are mainly focused on public security concerns, while few provisions are about personal data protection [28]. Data subject rights granted by these relevant laws are much narrower than those granted by the provisions in the GDPR.

It is important to note that data protection laws are generally lacking in low-income countries, and many have not promulgated privacy laws. According to the United Nations Conference on Trade and Development, 19% of low-income countries have no privacy legislation, and just 10% have developed a draft of their intended policies [29]. This privacy gap brings significant legal barriers to transnational data transfer and sharing.

### Some Jurisdictions Allow for Transnational Data Transfer, While Others Do Not

Many domestic data protection regulations have already established conditions for transnational data transfer, and they are varied among jurisdictions. Consequently, the cross-border communication and transmission of epidemic contact tracing data will inevitably encounter legal challenges.

In China for example, data must undergo an internal security assessment that adheres to the regulations established by state network departments before data can be transferred outside the country. According to the Security Assessment Measures for Cross-Border Transfer of Personal Information (Draft for Comment) issued in 2019, network operators shall apply security assessments for the cross-border transfer of personal information to local cyberspace administrations at the provincial level before the personal information leaves China [30].

In the European Union, the GDPR imposes strict limitations on using a third party (ie, a country outside the European Economic Area or an international organization) to process data transfers (chapter V of the GDPR) [31], as this is likely to happen in international traveling. Transference can only take place if the law applicable by the third party “ensures an adequate level of protection”, as confirmed by an adequacy decision taken by the European Commission (Article 45 of the GDPR) [31], or “if the controller or processor has provided appropriate safeguards” regarding data safety, the protection of the subject’s rights, and the existence of adequate remedies (Article 46 of the GDPR) [31]. Apart from these 2 cases, only the specific and exceptional conditions laid down in Article 49 of the GDPR remain [31]. This specific set of scenarios for data transfer might hamper the operationality of non-European Union contact tracing apps. Contact tracing apps used in a country where data security protection does not meet the European Union’s standards cannot obtain the infection risk information of travelers entering European Union member states. Hence, the destination country is not able to determine whether a traveler has already been infected before entering the country or has become infected after entry.

In some countries, data collected by contact tracing apps are not allowed to be transferred outside of the country. For example, in Australia, the Privacy Amendment (Public Health Contact Information) Act 2020 prohibits a person from disclosing data collected by contact tracing apps to another person outside of Australia [32]. The Australian COVIDSafe app also contains statements in the Terms of Service section that describes how all data collected by the government through the app are kept in Australia and cannot be transferred out of the country [33].

At the international level however, no mechanism enables the sharing of data essentials for pandemic control. Region-based contact tracing apps work as a technologic Tower of Babel, as they operate independently from one another and are not mutually recognized. The isolation of each app leaves a monitoring gap for international travel, which leads to real challenges for global pandemic control.

## Minimizing the International Health Risks Posed by This Technologic Tower of Babel

### Possible Options for Destroying This Technologic Tower of Babel

In April 2020, both the European Parliament [34] and the European Commission [35] called on member states to work together to fight the pandemic. In June 2020, the European Data Protection Board released a statement regarding the impact of data protection on the interoperability of contact tracing apps within the European Union [36]. Several months later, in October 2020, a European Union-wide system for contact tracing app interoperability was launched, and the first group of contact tracing apps (ie, Germany’s Corona-Warn-App, Ireland’s COVID tracker app, and Italy’s Immuni app) were linked to the system [37]. However, a broader consensus is required for the international community, since a common European approach will only solve the problem within the European Union.

The World Health Organization (WHO) has established ethical guidelines for directing member parties in the use of contact apps for the COVID-19 pandemic control [38]. Recently, the WHO Emergency Committee has also been actively working on the development of public health tools that can help member states deal with the pandemic-related risks involved with the gradual resumption of international travel [39]. These recommendations provide evidence-informed guidelines that member states can refer to when developing policies for pandemic control. To date, however, no international instrument has been established to address the conflicts of laws regarding data protection and privacy issues between member states. In this regard, we advocate that the global community should use the COVID-19 crisis as an opportunity for developing a mechanism that can facilitate the transnational sharing of data among countries during global public health emergencies. The biggest challenges will be reaching an agreement between countries and determining methods for enforcing such a mechanism.

Not long ago, the Global Outbreak and Response Network, which worked under the WHO, established a global data collection and sharing platform, Go.Data, which was used in South African countries for Ebola surveillance [40]. Ideally, a similar model that includes a large array of countries—potentially, all the affected ones—could be put in place for the COVID-19 pandemic. However, practical and legal impairments prevent this possibility. At most, we can aspire to create an open-access platform with aggregated data (ie, anonymized data) so that international travelers, institutions, and governments around the world can use the data to track the trends of infected persons and inquire about the risk of epidemic infection in a country or region.

Another bold proposal is a common contact tracing app that is accepted by all countries and made mandatory for international travelers, to help authorities track the flow of potentially infected international travelers. This international contact tracing app will fill the supervisory vacuum for international traveling, providing governments with real-time data to prevent the international spread of disease. Owing to the global citizenship obligation, international travelers should be required to install this tracing app [41]. This specific model for a contact tracing app would result from a common agreement. Governments should agree on a technological model for this app (Table 1). In any case, only the data necessary to perform the specific task assigned to this app should be collected. Such data should not include the biometric information of users, and the data collected should only be used for monitoring epidemic risk. As Google and Apple have worked together to promote contact tracing technology that allows communication between smartphones using iOS and Android systems via Bluetooth, this technologic progress may provide an option for minimizing international health risks. Based on Bluetooth and users’ self-reports, this common contact tracing app would notify app users who have had close contact with a COVID-19–positive app user during international travel in the past 14 days.

### Reality Check

**Figure 1:**
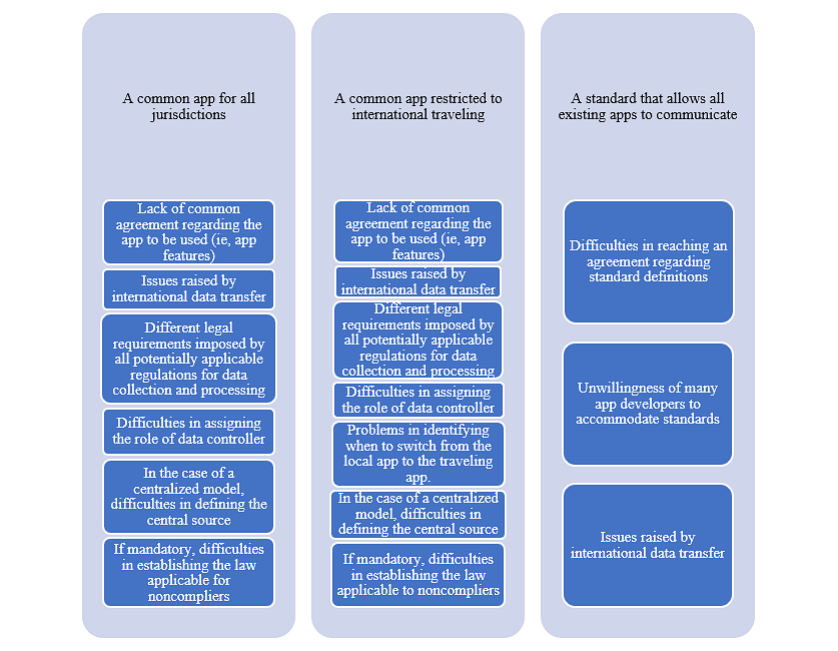
Potential solutions (light blue box) to the technologic Tower of Babel and barriers to implementing these solutions (dark blue box).

Implementing any of these measures would not be easy in jurisdictions with rather stringent privacy laws. Each government has to accept a common data collection, processing, and transfer regime that is exclusively applicable to COVID-19 contact tracing apps as an exception to their respective national laws on privacy. This equates to a uniform privacy policy for all contact tracing app users (Figure 1).

Even with the leadership of the WHO and the menace of a global pandemic, the goal of fulfilling these measures is difficult to achieve. Governments will not easily give up the power to decide which contact tracing apps would be used in their territory and which laws should govern these apps (ie, their own laws). Even if they participate in such an international project, without global consensus on a legal framework for data collection, processing, and transfer, it is very unlikely that all countries can agree on a single model for contact tracing apps (Figure 1). Moreover, issues raised by international data transfer are another real challenge for achieving the interoperability of contact tracing apps. In particular, a recent decision made by the Court of Justice of the European Union (CJEU) indicates the legal challenges for transnational personal data transfer due to different data protection standards. In July 2020, the CJEU announced that the US-EU Privacy Shield, the personal data transfer arrangement between the United States and European Union, cannot provide sufficient data protection, and is therefore invalid [42]. This decision will dramatically impact personal data flows between the United States and European Union and will inevitably hamper the communication between contact tracing apps used in the 2 jurisdictions (Figure 1).

## Conclusion

Contact tracing apps can be powerful mechanisms for handling the pandemic. However, their benefits will be lost if a patchwork system of noncommunicating apps becomes normal. If governments and app developers do not act cooperatively and strategically, the global community will be left to navigate dozens of apps operating under different protocols, data models, and legal rules, ultimately hampering the effort to control the COVID-19 pandemic.

Developing an international instrument that regulates data transfer between different countries’ contact tracing apps is a challenging goal. The negotiation for such an instrument demands strong leadership and consistent efforts from the entire international community. Due to the reopening of international travel, we face the increased risk of future waves of COVID-19. Therefore, collaborative actions should be taken to minimize the risk of COVID-19 spread in international travel and transport. The international community should start working together to establish a framework that enables data sharing and data transfer among different contact tracing apps, thereby breaking this technologic Tower of Babel.

In spite of the existing difficulties, the goal of implementing these solutions is still worth pursuing, as it is for the common good for all human beings. The isolation of contact tracing apps will not contain the international spread of infectious diseases. Instead, their isolation will result in the creation of a useless technologic Tower of Babel. The international community should be prepared for the next global pandemic crisis.

At present, it is crucial for all of us global citizens to collaborate with the common goal of establishing mechanisms that fix the current fragmentation of contact tracing systems and enable effective global epidemic surveillance. Only time will tell if national governments will primarily protect their power and regulations—which they feel is their sovereign right—under the price of paralyzing international traveling and jeopardizing our common safety. As it happened in biblical accounts, this technologic Tower of Babel will throw us apart.

## Abbreviations

API: application programming interface
CCPA: California Consumer Protection Act
CJEU: Court of Justice of the European Union
EPHIC: Epidemic Prevention and Health Information Code
FIPPA: Freedom of Information and Protection of Privacy Act
GDPR: General Data Protection Regulation
HIA: Health Information Act
HIPPA: Health Insurance Portability and Accountability Act
WHO: World Health Organization
