# Correction: COVID-19 Contact Tracing Apps: A Technologic Tower of Babel and the Gap for International Pandemic Control

**DOI:** 10.2196/26239

**Published:** 2020-12-04

**Authors:** Li Du, Vera Lúcia Raposo, Meng Wang

**Affiliations:** 1 Faculty of Law University of Macau Macau, SAR China; 2 Faculty of Law University of Coimbra Coimbra Portugal

In “COVID-19 Contact Tracing Apps: A Technologic Tower of Babel and the Gap for International Pandemic Control” (JMIR Mhealth Uhealth 2020;8(11):e23194) the authors noted one error.

This article was inadvertently published with the incorrect country code for the phone number of the Corresponding Author. The phone number was originally published as “86 88224733”. This has been corrected to “853 88224733”.

The correction will appear in the online version of the paper on the JMIR Publications website on December 7, 2020, together with the publication of this correction notice. Because this was made after submission to PubMed, PubMed Central, and other full-text repositories, the corrected article has also been resubmitted to those repositories.

